# Nebulized antibiotics for ventilator-associated pneumonia: misleading analysis and interpretation of the data

**DOI:** 10.1186/s13054-015-0952-3

**Published:** 2015-06-15

**Authors:** Wan-Jie Gu

**Affiliations:** Department of Anesthesiology, Affiliated Drum Tower Hospital, Medical College of Nanjing University, 321 Zhong Shan Road, Nanjing, 210008 Jiangsu China

I read with interest the recent systematic review of nebulized antibiotics for ventilator-associated pneumonia [1]. I congratulate and applaud Zampieri and colleagues’ important work, but several important issues should be noted.

First, there is one important methodological issue with the review. The data from randomized controlled trials and observational studies were inappropriately pooled together, which goes against the precept of pooling studies with similar design. In fact, the pooling of data from randomized controlled trials and observational studies is only recommended for the assessment of harm/adverse effects. Thus, results from randomized controlled trials and results from observational studies should be separately pooled.

Second, two of the included observational studies were case–control studies [2, 3], and therefore using the relative risk as the summary statistic is improper. Instead, the odds ratio should be used as the summary statistic to pool data from observational studies.

Third, the authors used the standardized mean difference as the summary statistic for the continuous variables. As we know, the standardized mean difference is unitless because it is a relative, rather than an absolute, measure of effect. Instead, the authors should have used the mean difference.

Last, Kalin and colleagues’ study should be excluded from the review. This study does not state why patients received inhaled colistin and how many patients received a high, normal or low dose in the inhaled plus intravenous colistin group and the intravenous colistin group [4]. Inadequate allocation may thus exist, resulting in a significant potential for selection bias. Inclusion of this study leads to significant heterogeneity, as shown in the forest plot for clinical cure.
